# Public perceptions and attitudes of the national project of bio-big data: A nationwide survey in the Republic of Korea

**DOI:** 10.3389/fgene.2023.1081812

**Published:** 2023-02-23

**Authors:** Ji Hyun Yang, Hannah Kim, Ilhak Lee

**Affiliations:** ^1^ Division of Medical Law and Ethics, Department of Medical Humanities and Social Sciences, Yonsei University College of Medicine, Seoul, South Korea; ^2^ Asian Institute for Bioethics and Health Law, Yonsei University, Seoul, South Korea

**Keywords:** ELSI, big data, survey, data sharing, precision medicine

## Abstract

**Background:** The National Project of Bio-Big Data (NPBBD) is a South Korean bio-big data collection project, expected to include health, genomic, and lifelog data of one million Koreans. The Ethical, Legal, and Social Implications study is a parallel study active since 2020. As part of the study, a public survey was conducted to evaluate public attitudes towards engagement schemes, such as public committees and web portals for communication between the public and researchers.

**Methods:** An online survey was conducted from March 3–9, 2021, using structured questionnaires addressed to 1,000 adults aged 20–59 years.

**Results:** Several respondents reported a positive attitude towards participation (43.6% “somewhat,” 14.3% “definitely”), whereas approximately one-third (36.5%) reported a neutral attitude. Positive factors that may affect the willingness of the respondents to participate included receiving health information (25.1%), contributing to research on cancer and rare diseases (21.9%), and advancing personalized medicine (21.5%). Conversely, negative factors were mainly associated with concerns regarding the risk of data leakage (22.8%), discrimination (21.1%), lack of information (13.5%), possibility of knowing the risk of being diagnosed with an incurable diseases (12.5%), and possibility of using data in industry (11.3%). In terms of project governance, respondents tended to recognize the importance of public participation in incorporating public opinion into the project design.

**Conclusion:** These results have implications for the participant recruitment process, public engagement strategies, and the scope of user (academics/industry, domestic/overseas) accessibility to the database.

## 1 Introduction

Big data is a key resource for research and development in the healthcare field, as well as in other areas of research (Mehta et al., 2019). Bio-big data, which integrates clinical-medical information, genomic information, and lifelogs, can contribute to the development of data analysis and processing technology, which can provide new insight for the prevention and treatment of diseases (Hamburg and Collins, 2010; Ashley, 2016; Prosperi et al., 2018; Suwinski et al., 2019). Construction and utilization of a bio-big data platform are essential for precision medicine in determination of the optimal healthcare and treatment for individuals (Obermeyer and Emanuel, 2016; Agrawal and Prabakaran, 2020). In fact, several national bio-big data projects are already in operation, including the All-of-us Research Program in the US and the 100,000 Genome Project in the UK (Collins and Varmus, 2015; Peplow, 2016; Barwell et al., 2018; Denny et al., 2019). These projects not only collect information and samples from a large number of participants, but they also plan to provide each participant with the results obtained from their analyses, forming a virtuous cycle by sharing the objectives achieved by the bio-big data project with individuals and society as a whole.

The Korean National Project of Bio-Big Data (NPBBD) is a public participation project aiming to recruit more than one million participants to build a database, in which, Korean health and genetic data are collected through voluntary participation, managed on a safe platform, for use by qualified researchers. The data collected includes clinical and genomic data and, in some cases, lifelogs. This project has been operating in its pilot stage since 2020 and has been managed by four government agencies and 16 hospitals nationwide. Data collected through this project will be permanently stored and used, unless the withdrawal of consent is explicitly indicated.

The establishment of such large-scale integrated bio-big data is expected to impact society in general, both in a positive and negative way. Utilization of bio-big data is expected to result in the progress of healthcare by enabling the investigation of the risk factors associated with cancer or rare diseases, which would facilitate more effective and efficient healthcare delivery (Mehta et al., 2019); however, bio-big data also harbors the potential issues of breach of data privacy, misuse and abuse of personal data, and social misunderstanding of personal traits and social values (Kaye, 2012; Shabani et al., 2014; Sankar and Parker, 2017; Oliveri et al., 2018; Vayena and Blasimme, 2018; Hallowell et al., 2019). As ethical, legal, and social issues may arise from the size, type, and sensitivity of data collection, we are conducting an Ethical, Legal, and Social Implications (ELSI) research program to identify and respond to these concerns; the NPBBD has established an independently organized and managed ELSI committee. For this program, the authors conducted a survey, and the results will be used as evidence to be utilized by future ELSI committee operations, project governance composition plans, and related policies.

The objective of this study was to identify the targets of the NPBBD and manage short- and long-term strategies for participant recruitment, public communication, and data sharing policies. We aimed to: 1) assess the perceived needs and expectations of the government to implement a large-scale data sharing platform and identify the factors that may impact the willingness of respondents to participate in this project, 2) respondents’ willingness to participate in the governance committee as participant representatives, and 3) evaluate to what extent participants were willing to agree to data sharing.

## 2 Materials and methods

### 2.1 Survey methods

This survey was conducted online using structured questionnaires and involved 1,000 adults aged 20–59 years. Participants were recruited based on the statistics of resident registration of the Ministry of Interior and Safety at the end of January 2021. The sampling method was a simple proportional allocation based on region, gender, and age; a professional survey company, DFINE ((주)디파인앤코) was responsible for administering the survey. The estimated margin of error was ± 3.1%, and the survey was conducted online from March 3–9, 2021 (7 days).

### 2.2 Questionnaire development

The questionnaire used in this study was developed in accordance with the previous study by Kim et al. (2020). The questionnaire was written in Korean and included 20 main questions with single-select or multiple-select answers or with a five-point scale on answer sheets. It also included five items on social/demographic variables. The Korea Disease Control and prevention Agency (KDCA), which co-manages the NPBBD with other governmental agencies and supports the ELSI program of the NPBBD, provided a brief introduction to the project, including the project objectives, types, and scale of data to be collected; data-sharing methods; and study areas. The respondents were asked about their: 1) awareness of bio-data, big data, biobanking, and the NPBBD; 2) expectations towards the NPBBD; 3) attitudes toward participating in the NPBBD; 4) decision-making factors for participation; 5) preferred ways to receive information about the NPBBD; 6) willingness to participate in public representative committees; 7) return of results preferences; and 8) data sharing and usage domain preferences. In addition to sociodemographic information, we added variables related to familiarity with the digital environment and other research participation experiences. After completing the survey, the respondents were rewarded virtual points that could be redeemed for cash.

### 2.3 Ethical approval

The survey was approved by the Institutional Review Board (IRB) of the Yonsei University Health System (approval number: Y-2020-0234). According to the Bioethics and Safety Act, the requirement for written consent was waived by the IRB because, due to the nature of the survey form, the respondents had to read the survey information before starting the survey and, therefore were perceived to have agreed to participate in the survey. The survey was also designed to allow participants to withdraw their participation at any time during or after the survey.

### 2.4 Statistical analysis

Data were analyzed using IBM SPSS Statistics for Windows version 22. Missing data were excluded from the online survey. Sociodemographic characteristics of the respondents, including age, gender, household income, and education were analyzed. Statistical significance was set at *p* < 0.05.

## 3 Results

### 3.1 Respondents

Sociodemographic and digital environment familiarity characteristics of the study population are shown in Table 1. A total of 19,023 individuals were invited to participate in the survey by email, and 1,785 people were enrolled from the website. Among them, 1,000 fully responded, resulting in an invitation response rate of 5.3% and an access response rate of 9.3%.

**TABLE 1:** Sample description (n = 1,000). Participants’ willingness to participate in the NPBBD according to prior knowledge of the NPBBD and demographic variables.

Variable	Categories	Total	Unwilling	Unsure	Willing	*p*-value^a^
		N	N (%)	N (%)	N (%)	
All		1000	56 (5.6)	365 (36.5)	579 (57.9)	
NPBBD Knowledge	Heard of it and know it well	112	3 (2.7)	13 (11.6)	96 (85.7)	<0.001
	Heard of it but do not know it well	452	8 (1.8)	136 (30.1)	308 (68.1)	
	Have not heard of it	436	45 (10.3)	216 (49.5)	175 (40.1)	
NPBBD Expectations	Low	4	3 (75.0)	1 (25.0)	0 (0.0)	<0.001
	Middle	137	17 (12.4)	90 (65.7)	30 (21.9)	
	High	859	36 (4.2)	274 (31.9)	549 (63.9)	
Gender	Male	514	30 (5.8)	169 (32.9)	315 (61.3)	0.050
	Female	486	26 (5.3)	196 (40.3)	264 (54.3)	
Age	20–29	224	19 (8.5)	75 (33.5)	130 (58.0)	0.072
	30–39	223	9 (4.0)	96 (43.0)	118 (52.9)	
	40–49	272	17 (6.3)	99 (36.4)	156 (57.4)	
	50–59	281	11 (3.9)	95 (33.8)	175 (62.3)	
Education	high school	135	7 (5.2)	56 (41.5)	72 (53.3)	0.722
	College	756	42 (5.6)	268 (35.4)	446 (59.0)	
	Graduate school and beyond	109	7 (6.4)	41 (37.6)	61 (56.0)	
Household income (Monthly/KRW)	≤3.99 million	395	29 (7.3)	175 (44.3)	191 (48.4)	<0.001
	4.00–5.99 million	325	11 (3.4)	113 (34.8)	201 (61.8)	
	≥6.00 million	280	16 (5.7)	77 (27.5)	187 (66.8)	
Private health insurance user	Yes	774	37 (4.8)	265 (34.2)	472 (61.0)	0.001
	No	226	19 (8.4)	100 (44.2)	107 (47.3)	
Wearables user	Yes	325	11 (3.4)	85 (26.2)	229 (70.5)	<0.001
	No	675	45 (6.7)	280 (41.5)	350 (51.9)	
Health apps user	Yes	550	18 (3.3)	166 (30.2)	366 (66.5)	<0.001
	No	450	38 (8.4)	199 (44.2)	213 (47.3)	
Social media service user	Yes	913	46 (5.0)	314 (34.4)	553 (60.6)	<0.001
	No	87	10 (11.5)	51 (58.6)	26 (29.9)	

a χ^2^ Analyses

The particpants’ age (22.4% in 20 s, 22.3% in 30 s, 27.2% in 40 s, 28.1% in 50 s) and gender (51.4% male and 48.6% female) were approximately evenly distributed. The distribution of monthly household income in Korean Won (KRW) was ≤ 3,990,000, 39.5%; 4,000,000–5,990,000, 32.5%, and ≥6,000,000, 28%. Additionally, educational level was distributed as follows: high school graduates, 13.5%; college attending or graduate, 75.6%; and graduate school and beyond, 10.9%.

Although most Koreans are covered by the National Health Insurance system, most (77.4%) of the participants had private health insurance. Of the respondents, 32.5% used wearable devices (e.g., Fitbit, Apple watch, Galaxy watch), 55% used health apps, and 91.3% used social media services. The average number of hospital visits per year was four in 2020. According to national statistics, the number of days of annual visits to medical institutions per person in Korea was approximately 21 days in 2019 and approximately 19 days in 2020. Considering that COVID-19 occurred in 2020, the number of hospital visits of survey respondents may have been slightly lower than usual (Oh et al., 2021).

### 3.2 Awareness of bio-data and biobanking

**FIGURE 1:**
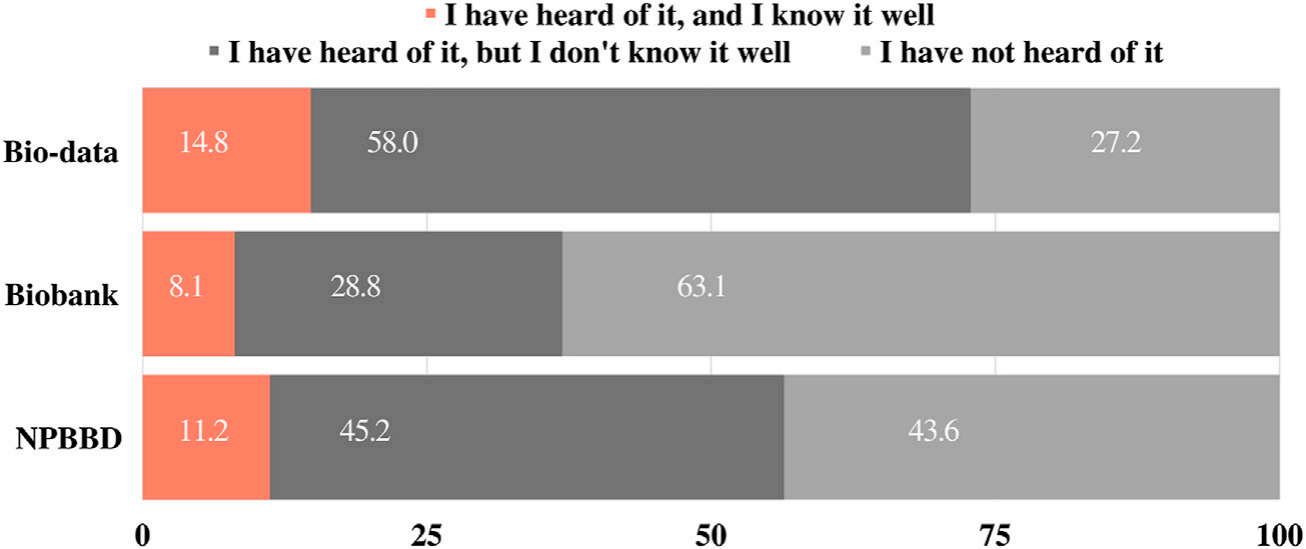
Awareness of bio-data, biobank, and the NPBBD, respectively.

Before asking about project awareness, we asked 1) “Have you heard of “bio-data” or “big data in healthcare”?” and 2) “Have you heard of a “biobank”?” as the preliminary questions. The response options were “a) I have heard of it, and I know it well,” “b) I have heard of it, but I do not know it well,” and “c) I have never heard of it.” Approximately 14.8% of respondents reported that they knew “bio-data” or “big data in healthcare” well, and 8.1% of respondents reported that they knew “biobanking” well. Most respondents (58%) said that they had heard of “bio-data” or “big data in healthcare,” but did not know it well, and notably, 61% had not heard of “biobanking” (Figure 1).

### 3.3 Awareness of the NPBBD

Participants were asked whether they had heard of the NPBBD and were given the same response options as those provided above. Of the respondents, 11.2% answered “I have heard of it, and I know it well,” 45.2% answered “I have heard of it, but I do not know it well,” and 43.6% answered “I have not heard of it” (Figure 1).

Among the 564 respondents who had heard of the NPBBD, 62.9% (n = 355) had learned about it through the Internet, 37.8% (n = 213) from television, 21.6% (n = 122) from magazines and newspapers, 20.7% (n = 117) from medical institutions, and 16.1% (n = 91) from people around them, such as friends and family.

### 3.4 Expectations for the NPBBD

**FIGURE 2:**
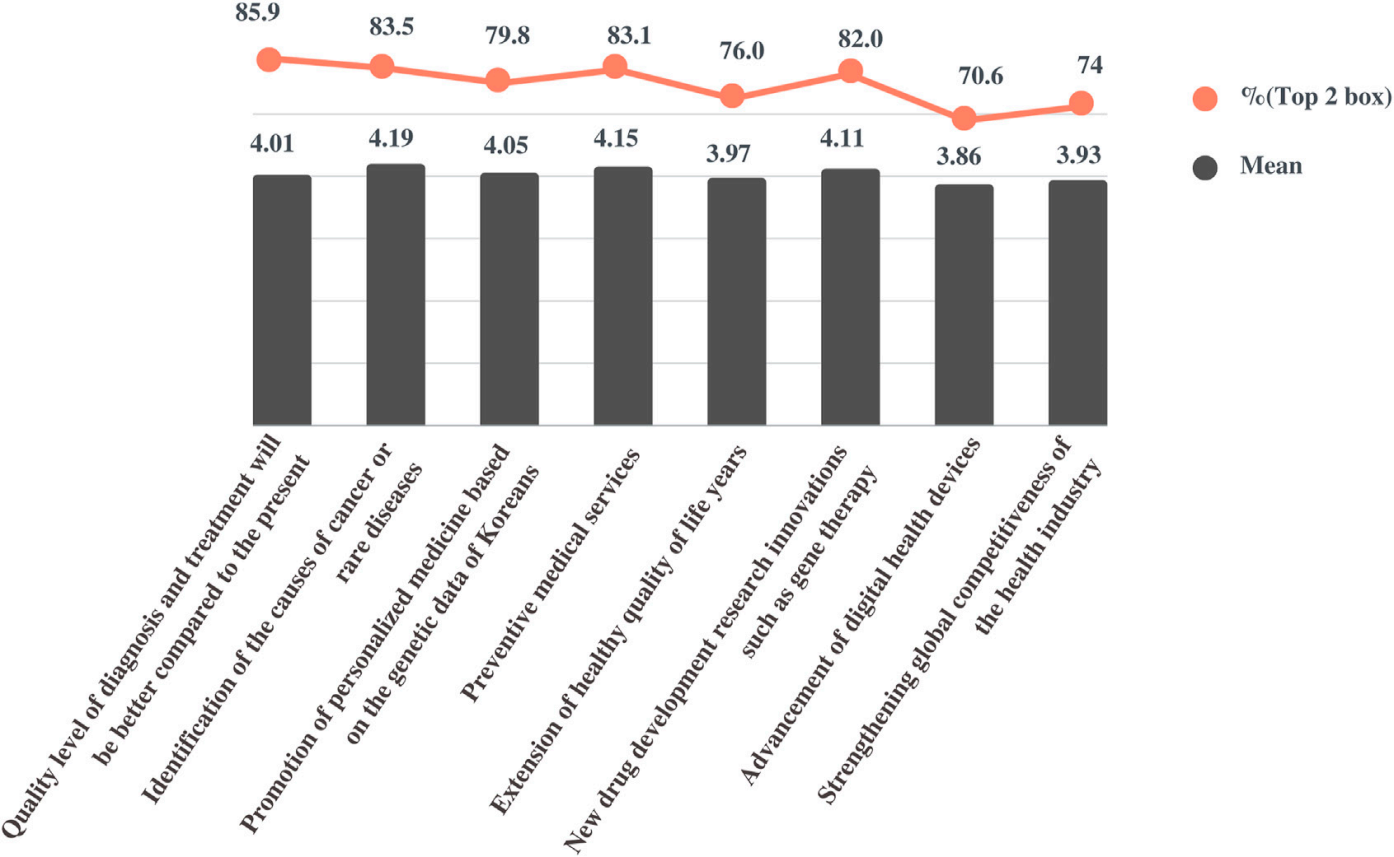
Perceived importance and effectiveness of the NPBBD. This figure shows the mean score and top two box score of the responses based on a five-point scale for the respective questions.

Participants were asked to describe the extent to which they agreed with the importance of the NPBBD in obtaining its expected outcomes. Participants were presented with seven items as expected effects of this project and five-point scale response options: “not important at all,” “of little importance,” “of average importance,” “important,” and “very important.” Responses were quantitated using a five-point scale (mean ± SD), and the percentage of respondents who indicated a top two box (T2B) was calculated. The overall response was positive: “identification of the causes of cancer or rare diseases” (4.19 ± 0.78, T2B = 83.5%), “preventive medical services” (4.15 ± 0.74, T2B = 83.1%), “new drug development research innovations such as gene therapy” (4.11 ± 0.72, T2B = 82%), “promotion of personalized medicine based on genetic data of Koreans” (4.05 ± 0.75, T2B = 79.8%), “extension of healthy quality of life years” (3.97 ± 0.76, T2B = 76%), “strengthening the global competitiveness of the health industry” (3.93 ± 0.75, T2B = 74%), and “advancement of digital health devices” (3.86 ± 0.77, T2B = 70.6%) (Figure 2).

Next, the participants were asked to describe to what extent they agreed with the statement, “the quality level of diagnosis and treatment will change compared with the present level.” The scales measuring the perceived effectiveness were quantified as follows: 1 “it will be much worse,” 2 “it will be somewhat worse,” 3 “it will stay the same,” 4 “it will be somewhat better,” and 5 “it will be much better.” On average (4.01 ± 0.57, T2B = 85.9%), respondents expected that the level of disease diagnosis and treatment would be improved compared with the present level if bio-big data were actively utilized in research through the NPBBD.

### 3.5 Attitudes toward participation in the NPBBD

**FIGURE 3:**
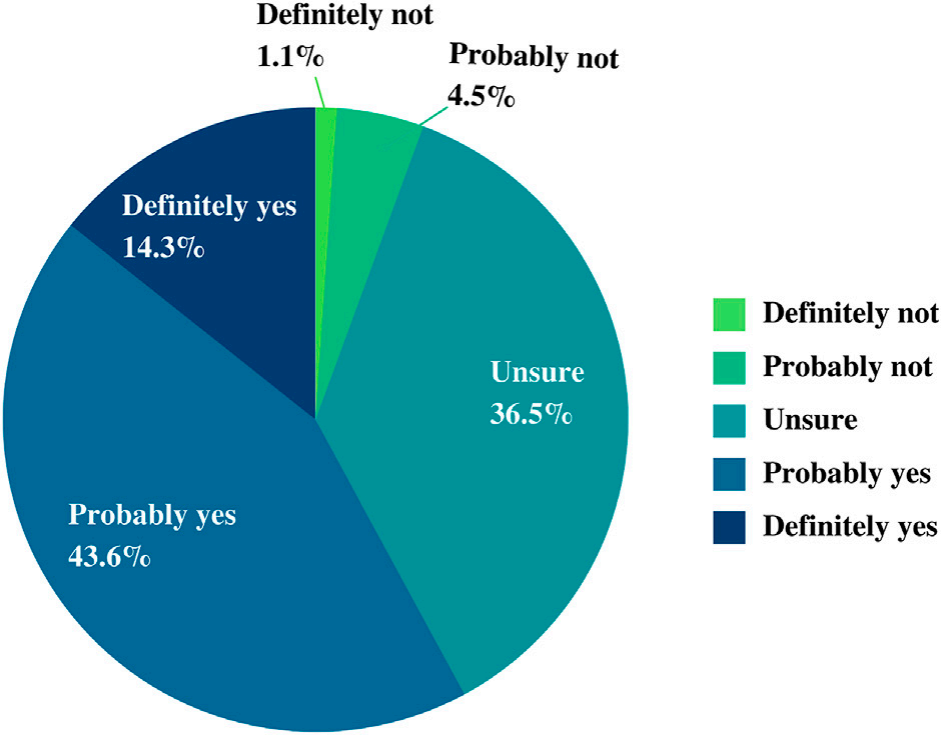
Willingness to participate in the NPBBD.

Participants were asked whether they would participate in the implementation of the NPBBD. In our survey, more than half of the respondents stated that they would “definitely or probably participate” (14.3% and 43.6%, respectively), 36.5% were “unsure,” and 5.6% said that they would “definitely or probably not participate” (1.1% and 4.5%, respectively) (Figure 3).

Regarding the characteristics of the respondents (Table 1), the intention to participate was higher in the group (85.7% and 68.1%, respectively) that had heard of this project than in the group (40.1%) that had never heard of it. Furthermore, the intention to participate in the project was relatively high in the group with experience using wearables (70.5%), health management apps (66.5%), and social media services (60.6%).

Compared with those who were willing to participate (Table 2), those who were unsure or unwilling were less likely to have heard of the NPBBD or used wearables, health apps, and social media services, and those who were unwilling were more likely to be in their 20 s. Table 2 also shows that people who were unsure of whether to participate were less likely to be male and more likely to be in their 30 s and have a low monthly household income.

**TABLE 2:** Multinomial logistic regression result for attitude toward participation in the NPBBD, with willing to participate as reference category (n = 579), associated with prior knowledge about NPBBD and demographic data. OR, odds ratio; LCI, lower 95% confidence interval; UCI, upper 95% confidence interval.

Variable	Category	Unwilling (n = 56)				Unsure (n = 365)			
		OR	LCI	UCI	*p*-value	OR	LCI	UCI	*p*-value
NPBBD Knowledge	Heard of it and know it well	0.122	0.037	0.401	0.001	0.110	0.059	0.202	<0.001
	Heard of it but do not know it well	0.101	0.047	0.219	<0.001	0.358	0.269	0.475	<0.001
	Have not heard of it	ref				ref			
Gender	Male	0.967	0.558	1.676	0.905	0.723	0.556	0.940	0.015
	Female	ref				ref			
Age	20–29	2.325	1.070	5.054	0.033	1.063	0.728	1.552	0.753
	30–39	1.213	0.488	3.019	0.677	1.499	1.038	2.164	0.031
	40–49	1.734	0.788	3.814	0.171	1.169	0.820	1.667	0.388
	50–59	ref							
Education	high school	0.847	0.282	2.550	0.768	1.157	0.683	1.962	0.588
	College	0.821	0.353	1.908	0.646	0.894	0.585	1.366	0.604
	Graduate school and beyond	ref				ref			
Household income (Monthly/KRW)	≤3.99 million	1.775	0.933	3.375	0.080	2.225	1.591	3.112	<0.001
	4.00–5.99 million	0.640	0.289	1.414	0.269	1.365	0.961	1.940	0.082
	≥6.00 million	ref				ref			
Private health insurance user	Yes	0.441	0.244	0.798	0.007	0.601	0.440	0.820	0.001
	No	ref				ref			
Wearables user	Yes	0.374	0.189	0.737	0.005	0.464	0.346	0.623	<0.001
	No	ref				ref			
Health apps user	Yes	0.276	0.153	0.495	<0.001	0.485	0.372	0.634	<0.001
	No	ref				ref			
Social media service user	Yes	0.216	0.098	0.476	<0.001	0.289	0.177	0.474	<0.001
	No	ref				ref			

### 3.6 Decision-making process for participation in the NPBBD

**FIGURE 4:**
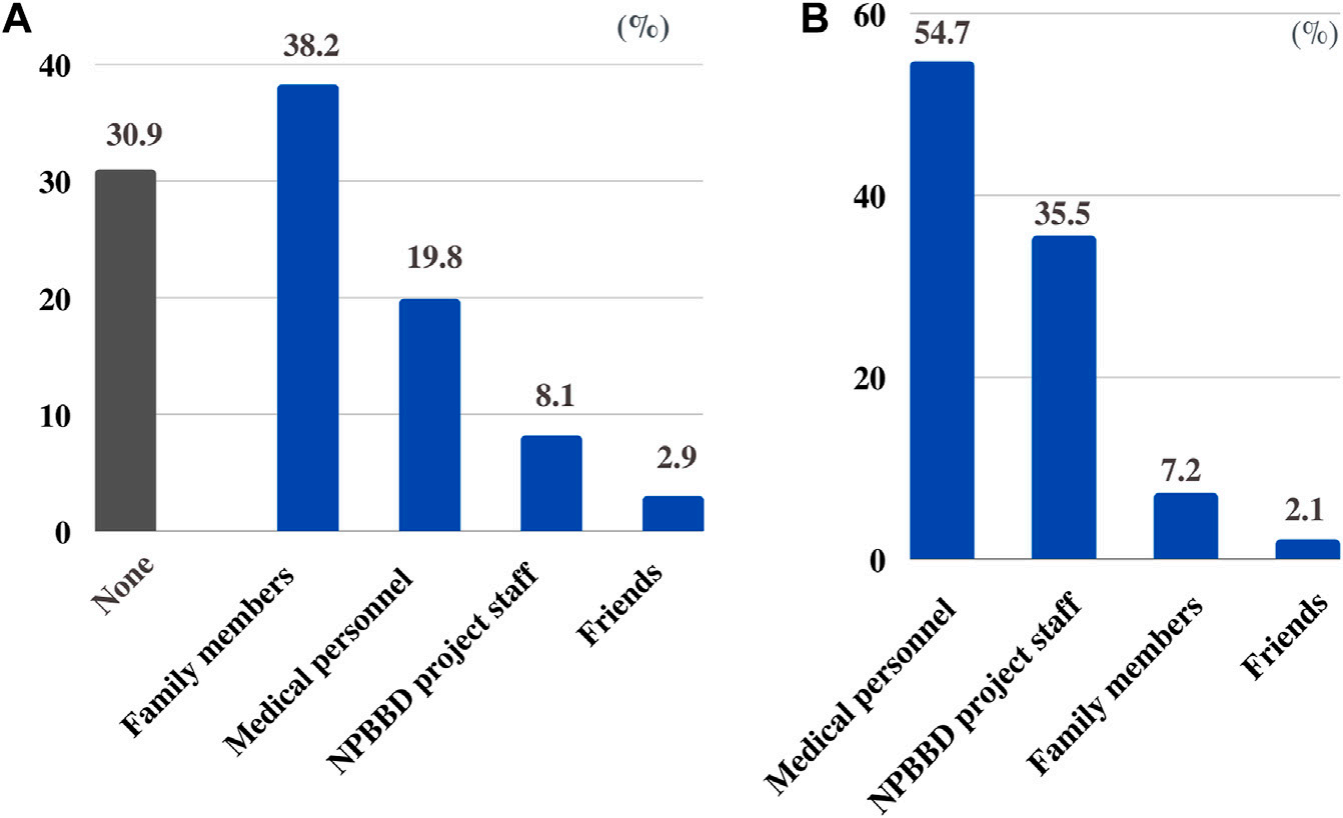
**(A)** With whom respondents want to consult regarding participation in the NPBBD. **(B)** From whom respondents want to be informed about the NPBBD when deciding whether to participate.

For the question “With whom do you want to consult regarding participation in the NPBBD?” “family members” (38.2%) was the highest choice, followed by “medical personnel including my attending physician” (19.8%), “NPBBD project staff” (8.1%), and “friends” (2.9%). However, 30.9% of the respondents chose “none,” indicating that they would prefer to decide on their own without consulting anyone (Figure 4A).

Conversely, regarding the question “From whom did they want to be informed about the NPBBD when deciding whether to participate?” most respondents wanted to hear from a “medical professional including my attending physician” (54.7%), followed by “NPBBD project staff” (35.5%), “family members” (7.2%), and “friends” (2.1%) (Figure 4B).

### 3.7 Factors affecting the decision to participate in the NPBBD

#### 3.7.1 Positive factors

The participants were presented with a list of hypothetical factors that would affect their potential willingness to participate in the NPBBD and asked to indicate which three of these had the most positive impact on them. The list of hypothetical benefits presented to the participants and the ratios for each answer were as follows: respondents (n = 3 × 100%), “receiving the benefits of providing health information” (75.2%), “contributing to identifying the cause of cancer and rare diseases” (65.7%), “contributing to the promotion of personalized medical services” (64.5%), “contributing to the competitiveness of the Korean bioindustry” (36.7%), “recommendations from medical personnel, such as attending physicians” (25.9%), “interest in government-driven projects” (17.3%), and “recommendation from family members” (14.3%). Supplementary Figure S1 also shows the ratio of answers for each item in the first to third answer boxes, respectively.

In our survey, the positive impact factors varied depending on the respondent age groups. For example, the proportion of respondents in their 30s who chose “recommendations from medical personnel” (32.3%) was higher than that in other age groups (20.3%–27.7%), and the proportion that chose “contribute to the identification of causes of cancer and rare diseases” (55.2%) was lower than that in other age groups (61.2%–74.7%). The proportion of people in their 20 s who chose to contribute to the “competitiveness of the Korean health industry” (44.2%) was higher than that of people in other age groups (33.1%–35.4%) (Supplementary Table S1).

#### 3.7.2 Negative factors

Then, the participants were presented with a list of hypothetical concerns that would affect their potential willingness to participate in the NPBBD and asked to indicate which three of these had the most negative impact on their participation. The list of hypothetical concerns presented to participants and the answered ratios of each of the items were as follows: respondents (n = 3 × 100%), “risk of data leakage” (68.5%), “discrimination concerns” (63.2%), “lack of information or consultation on the project” (40.6%), “possibility of knowing the risk of being diagnosed with an incurable disease” (37.5%), “lack of time” (33.9%), “the possibility of using data in the industry” (33.9%), and “opposition from family members” (21.9%). Supplementary Figure S2 also shows the answered ratio of each item in the first to third answer boxes, respectively.

The proportion of respondents who chose “lack of information or consultation on the project” in their 30 s (46.2%) and “opposition by family” in their 40 s (27.9%) was slightly higher than that of other age groups (ranges 36.8%–41.1% and 18.9%–21.1%, respectively). Conversely, those with a high school education had a higher rate of choosing “possibility of knowing the risk of being diagnosed with an incurable disease” (43.7%) compared with those with other educational backgrounds (32.1%–37.2%) (Supplementary Table S2).

### 3.8 Preferred ways to receive information about the NPBBD

The participants were presented with possible ways to receive information about the NPBBD (e.g., information about the enrollment process, number of enrolled participants, data access criteria for researchers, research lists, operation of the ethics committee, etc.), and asked to indicate their two most preferred methods. The responses were as follows: text messages or email (37.6%), NPBBD website (24.9%), postal mail (17.2%), social media (15.5%), and phone calls (4.9%) (Supplementary Figure S3).

We asked all respondents whether they would like to use a website on which they could check information about the NPBBD, and 85% of the respondents answered “yes.” That is, although the preferred means of communication were different for each age group, the use of websites was generally considered positive.

### 3.9 Attitudes toward public engagement activity

We asked all respondents whether they would like to participate in the “committee made up of patients, their families, and the general public.” Approximately half of the respondents answered “yes” (50.3%).

### 3.10 Preferences for return of results

**FIGURE 5:**
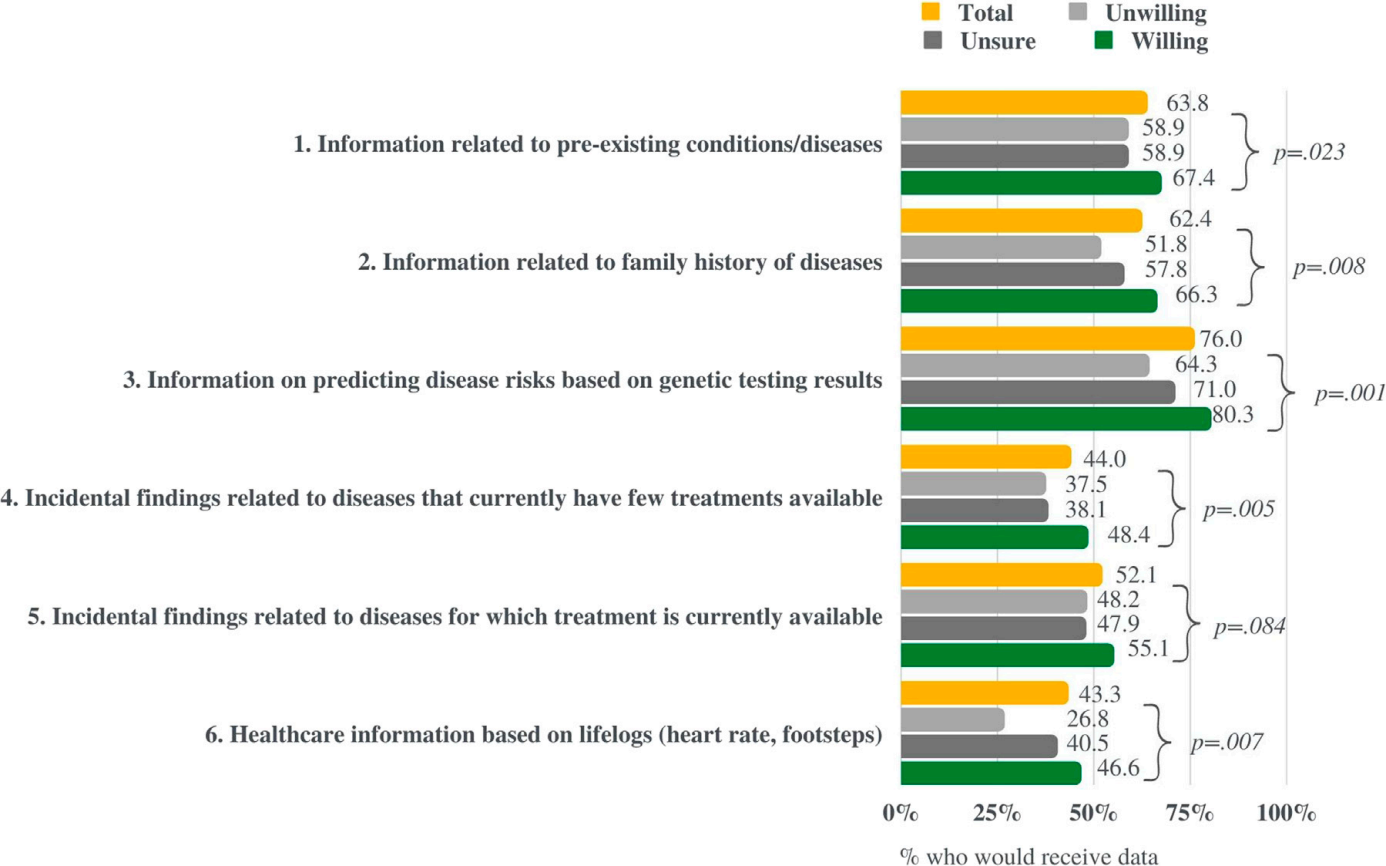
Respondents’ interest in different types of information the study could return to participants. This figure compares the results of those willing, unsure, and unwilling to participate in the NPBBD.

Respondents were asked “what type of information would you like to receive regarding your personal test results from the NPBBD? Please select everything you want to know” and presented with a list of options: “information on predicting disease risks based on genetic testing results,” “information related to pre-existing conditions/diseases,” “information related to a family history of diseases,” “incidental findings related to diseases that currently have few treatments available,” “incidental findings related to diseases for which treatment is currently available,” and “healthcare information based on lifelogs (heart rate, footsteps).” The results showed that “information on predicting disease risks based on genetic test results” was the most common choice (76%), followed by “information related to pre-existing conditions” (63.8%) and “family history” (62.4%) (Figure 5).

### 3.11 Attitude toward data sharing

#### 3.11.1 Willingness to provide medical information by purpose

**FIGURE 6:**
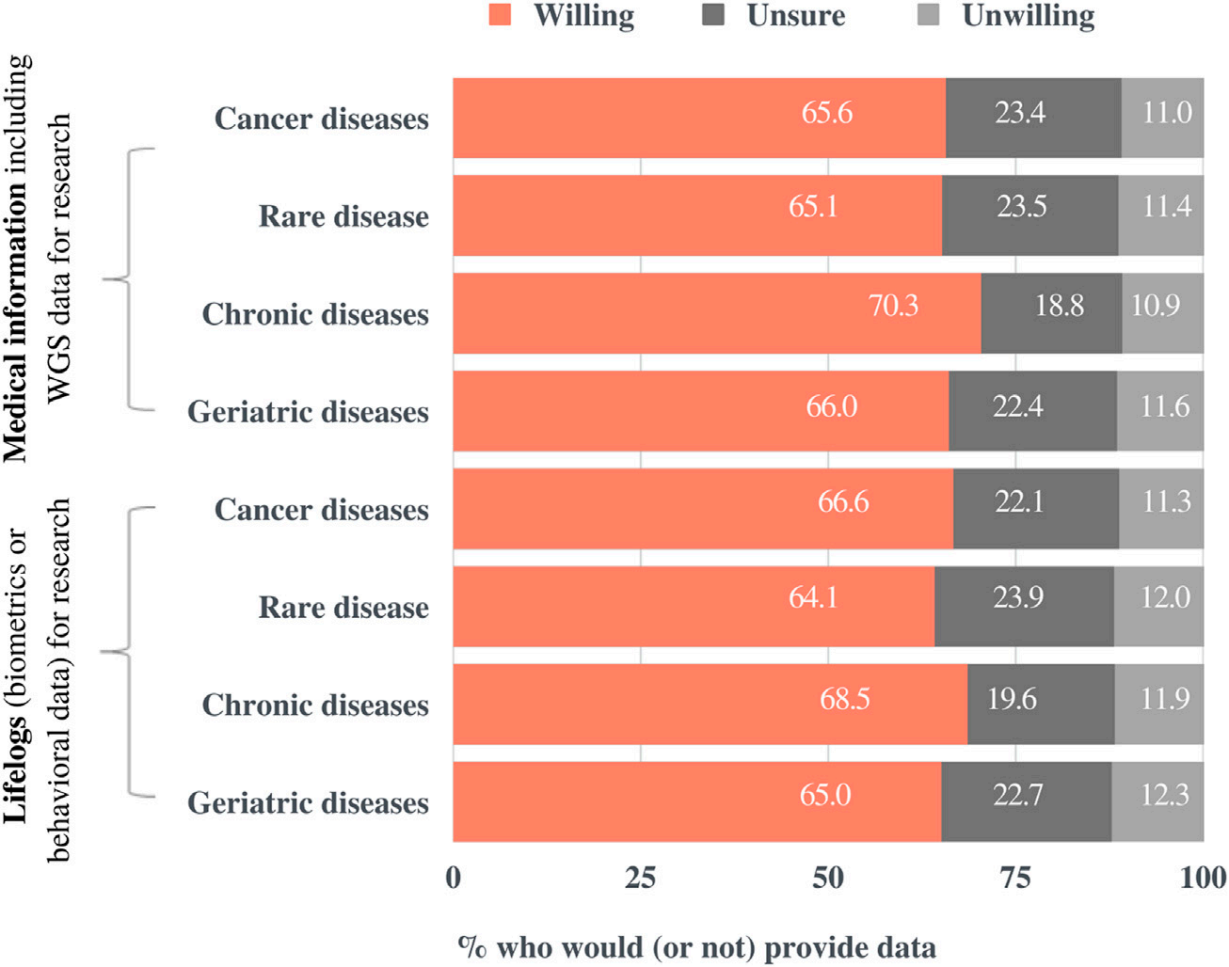
Preferences for sharing medical information for research purposes by research field. Participants were presented with four potential areas of study, two kinds of data, and three response options.

Figure 6 demonstrates the percentage of respondents that would share their medical information for each potential research purpose: “cancer,” “rare diseases,” “chronic diseases” (e.g., diabetes, high blood pressure, etc.), and “geriatric diseases” (e.g., dementia, Parkinson’s disease, stroke, etc). The response options were “Yes,” “Unsure,” and “No.” The respondents were then presented with the same fields of study and response options for their willingness to share biometrics or behavioral information, namely lifelogs. Although respondents were more likely to provide their data to the study of chronic diseases than that of rare diseases, the field of study did not have a significant impact on their intention to share the data. The slightly lower willingness to share information for rare diseases than that for other variables may originate from the demographic characteristics of the respondents, that is, most of them are laypeople with respect to health. Finally, no significant difference was observed in the response ratios between medical information and lifelogs.

#### 3.11.2 Willingness to provide medical information by user

**FIGURE 7:**
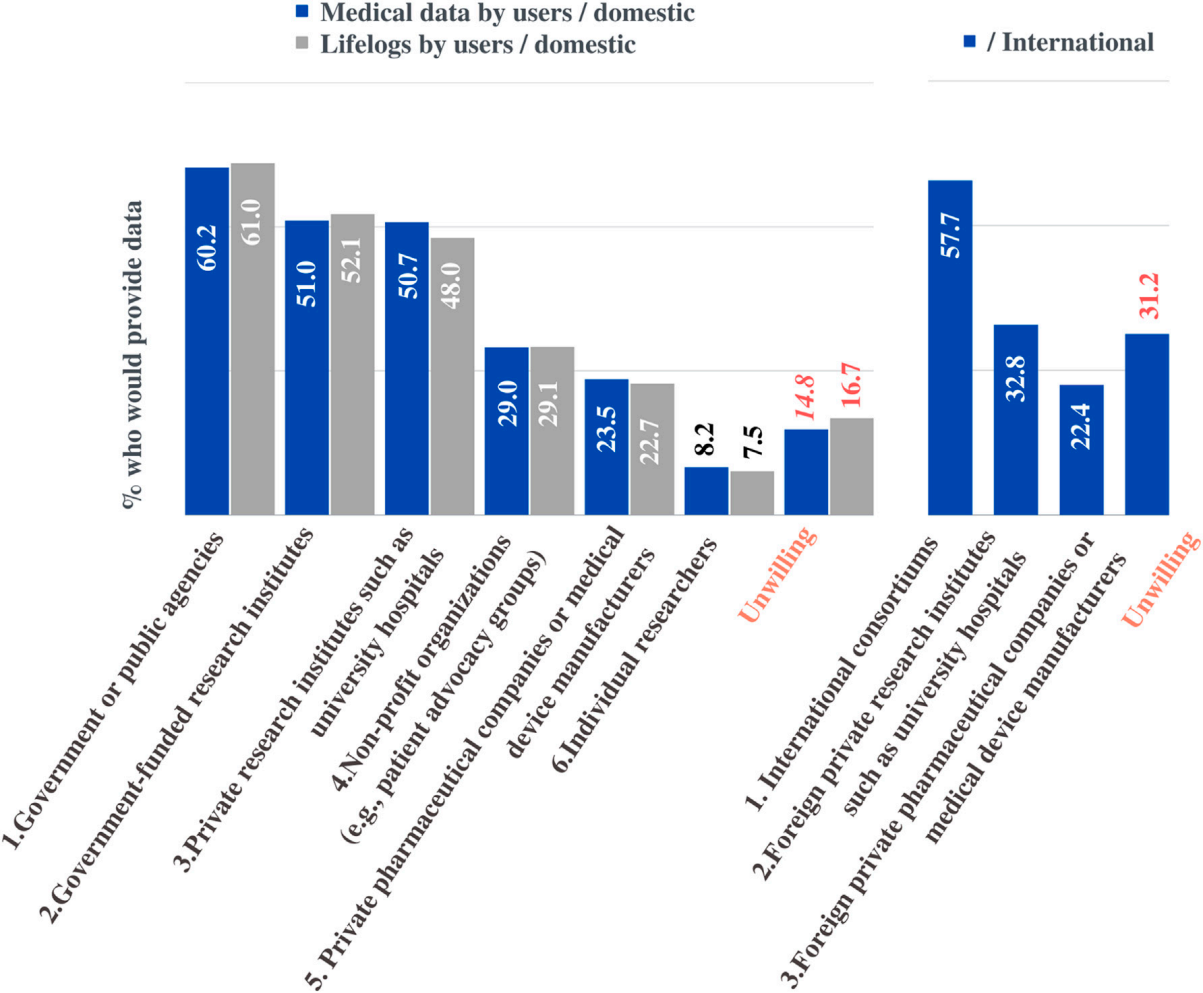
Preferences for sharing data for research purposes. The percentage of respondents who would share their medical information or lifelogs depending on data recipients.

Notably, the willingness to share data varied greatly depending on who would be the final user of the data (Figure 7). Participants reported that the use of medical information, including Whole Genome Sequencing (WGS) data, would be acceptable when accessed by the “government or public agencies” (60.2%), “government-funded research institutes” (51%), “private research institutes such as university hospitals” (50.7%), “non-profit organizations” (e.g., patient advocacy groups) (29.0%), and “private pharmaceutical companies or medical device manufacturers” (23.5%). Similarly, participants reported that the use of lifelog (biometrics or behavioral information) would be acceptable when accessed by the "government or public agencies” (61%), “government-funded research institutes” (52.1%), “private research institutes such as university hospitals” (48%), “non-profit organizations” (e.g. patient advocacy groups) (29.1%), and “private pharmaceutical companies or medical device manufacturers” (22.7%). Meanwhile, of the respondents, 14.8% and 16,7% indicated that they were unwilling to share their medical information and lifelog data, respectively. In general, participants preferred the public domain to the private sector and similar results were observed for international sharing.

Regarding international sharing of medical information, 57.7% of the respondents would be willing to provide information to an “international consortium,” 32.8% were willing to share with “research institutes such as foreign university hospitals.” The proportion of respondents who chose “foreign pharmaceutical companies and medical device manufacturers” (22.4%) was similar to that who chose “domestic private pharmaceutical companies and medical device manufacturers” (23.5%), but overall, they were more willing to agree for use in domestic situations.

## 4 Discussion

We found a slight gap between the level of expectations of the NPBBD and willingness to participate. Specifically, the expectations that this project will contribute to medical innovations are higher than the level of willingness to participate. Even among respondents who had positive expectations for the outcomes that can be achieved through the construction and utilization of bio-big data, their potential decisions on NPBBD participation were indecisive or negative in some cases. In this light, investigating perceived positive and negative factors for participation is an integral part of ELSI research to address the implications of operating the NPBBD and stimulate public discussion of these issues.

Regarding the awareness of the NPBBD, more than half of the participants (56.4%) answered that they had heard of the project, which was similar to the 58.2% reported by Kim et al. (2020). However, while 83.5% of the respondents said they would participate in the NPBBD in the study by Kim et al. (2020), this survey showed that only 57.9% would participate in the NPBBD. We hypothesized that this discrepancy may come from the tendency to make deliberate decisions or to withhold them. In our study, 36.5% of respondents answered that they were unsure whether they would be involved in the national project, but this trend was not found in the study by Kim et al. because the option was not available in their comparable question. Similar results of public attitudes toward participating in large-scale genomic studies were shown by Ishiyama et al. (2008) in Japan, in which 69.5% of respondents approved of the promotion of genomic studies related to medicine, and 29.3% were undecided.

Notably, in the questionnaire on positive motivation for participation, “receiving healthcare information” received the highest rating (75.2%). This confirms the results of an earlier study by Kim et al. (2020), which 80.2% of the respondents indicated “receiving health information” for important incentive in their decision about whether to participate in the proejct. Our findings also partially overlap those of the study in the US by Kaufman et al. (2016), which showed that the receipt of one’s own health information was the most important incentive for potential participation in the nationwide precision medicine initiative cohort. In our study, the next highest response rate on positive motivation was contributing to medical research for reasons such as “innovation of cancer and rare diseases treatment” (65.7%) or “personalized medical services” (64.5%). Overall, respondents found this project meaningful with respect to their contribution to disease treatment and prevention.

Conversely, for questions on negative motivation preventing participation, “data leakage concerns” (68.5%) and “discrimination concerns” (63.2%) were most frequently identified. Currently, the NPBBD securely maintains databases and grants access only to qualified researchers. Data is accessed in restricted areas with separate network systems, and proper research plans are essential for requesting access. Besides, article 46 of Bioethics and the Safety Act stipulates no person shall discriminate against any person on the ground of genetic information in employment, insurance, or any other social activity. Despite the existence of numerous physical, technical, and legal security measures, privacy concerns received high ratings. Given that public privacy concerns are common when personal data are collected and shared for multiple purposes (Middleton et al., 2019; Shah et al., 2019; Cheung et al., 2022), our findings confirm privacy is of such paramount importance that the NPBBD should protect. To alleviate privacy concerns, along with continuous efforts to implement practical measures that increase participants’ trust in privacy guarantees, precautionary policies that NPBBD follows need to be more actively communicated to the public.

We also elucidated the relationship between prior knowledge of the NPBBD and support for participation in it. Respondents who had heard about the project were more likely to be willing to participate in the NPBBD. Similarly, one’s familiarity with relevant knowledge of biodata is often examined alongside their attitude toward donating their own biodata. For example, in a survey of the English-speaking public’s attitudes by Middleton et al. (2019), familiarity with DNA or genomics was a key difference between those who were unsure about donation and those who were willing to donate. Additionally, in a survey of the German public’s attitude toward research biobanks by Bossert et al. (2018), concerning willingness to participate in biobanks, persons who had heard of biobanks before were more likely to be certain of their responses. Bossert et al. (2018) also suggested that increasing public awareness of biobanks may be a prerequisite for public engagement. These results imply that public relations activity is important in terms of facilitating supportive attitudes and for incorporating participants’ perspectives into the governance structure.

In addition, the preferred decision-making processes for participation and factors affecting them varied by respondents’ demographic profiles. Females (43.2% vs. males 33.5%) and older participants were more likely to consult their family members than males and younger participants, respectively. Among the respondents who said they would make decisions on their own without consulting others, the proportion of males (34.8%) was higher than that of females (26.7%). (Table 3). Pertaining to positively motivating factors for participation, the proportion of respondents in their 30 s who chose “recommendations from medical personnel” (32.3%) was higher than in other age groups (20.3%–27.7%). (Supplementary Table S1). In this regard, potential participants’ characteristics need to be taken into consideration when communicating with them. For instance, when utilizing official websites for general information sharing, various other means of communication, such as text messages, e-mail, postal mail, and social media should also be employed, depending on the content of the information provided and the characteristics of the recipient.

**TABLE 3:** Responses to “With whom do you want to consult regarding participation in the NPBBD?” according to various variables.

Variable	Category	1. None	2. Medical personnel	3. NPBBD staff	4. Family members	5. Friends	6. Other	Total	*p*-value^a^
		N (%)	N (%)	N (%)	N (%)	N (%)	N (%)	N	
All		309 (30.9)	198 (19.8)	81 (8.1)	382 (38.2)	29 (2.9)	1 (0.1)	1,000	
NPBBD Knowledge	Heard of it and know it well	36 (32.1)	18 (16.1)	23 (20.5)	34 (30.4)	1 (0.9)	0 (0.0)	112	<0.001
	Heard of it but do not know it well	124 (27.4)	111 (24.6)	31 (6.9)	171 (37.8)	15 (3.3)	0 (0.0)	452	
	Have not heard of it	149 (34.2)	69 (15.8)	27 (6.2)	177 (40.6)	13 (3.0)	1 (0.2)	436	
Gender	Male	179 (34.8)	100 (19.5)	49 (9.5)	172 (33.5)	13 (2.5)	1 (0.2)	514	0.008
	Female	130 (26.7)	98 (20.2)	32 (6.6)	210 (43.2)	16 (3.3)	0 (0.0)	486	
Age	20–29	66 (29.5)	56 (25.0)	28 (12.5)	66 (29.5)	8 (3.6)	0 (0.0)	224	0.001
	30–39	61 (27.4)	58 (26.0)	19 (8.5)	78 (35.0)	7 (3.1)	0 (0.0)	223	
	40–49	89 (32.7)	47 (17.3)	14 (5.1)	114 (41.9)	8 (2.9)	0 (0.0)	272	
	50–59	93 (33.1)	37 (13.2)	20 (7.1)	124 (44.1)	6 (2.1)	1 (0.4)	281	
Education	high school	39 (28.9)	23 (17.0)	6 (4.4)	63 (46.7)	4 (3.0)	0 (0.0)	135	0.164
	College	236 (31.2)	154 (20.4)	59 (7.8)	282 (37.3)	24 (3.2)	1 (0.1)	756	
	Graduate school and beyond	34 (31.2)	21 (19.3)	16 (14.7)	37 (33.9)	1 (0.9)	0 (0.0)	109	
Household income (Monthly/KRW)	≤3.99 million	147 (37.2)	79 (20.0)	26 (6.6)	125 (31.6)	18 (4.6)	0 (0.0)	395	0.001
	4.00–5.99 million	81 (24.9)	71 (21.8)	31 (9.5)	137 (42.2)	5 (1.5)	0 (0.0)	325	
	≥6.00 million	81 (28.9)	48 (17.1)	24 (8.6)	120 (42.9)	6 (2.1)	1 (0.4)	280	

a χ^2^ Analyses

Furthermore, the expressed preferences for sharing medical information or lifelogs for research purposes remarkably varied depending on who would use their data. Participants were more likely to provide their data to be shared with “government or public agencies” (>60%) than “non-profit organizations (e.g., patient advocacy groups)” (>29%) or “private pharmaceutical companies or medical device manufacturers” (>22%). Willingness to share their data with “government-funded research institutes” (>50%) and “private research institutes such as university hospitals” (>48%) was moderate. On the hand, there was no significant difference in willingness to provide medical information or lifelogs depending on which field of study their data would be shared with. Similar patterns were observed in research in the UK by Atkin et al. (2021), where fewer participants supported the use of their data by university researchers compared to NHS staff (84.9% v. 93.8%, respectively), and healthcare companies received even less supportive responses (65%). Other studies also found that respondents were particularaly less supportive if a commercial entity was the recipient of the health information (Milne et al., 2019; Barnes et al., 2020; Vidgen et al., 2020). Additionally, a survey of the European public by Shah et al. (2019) suggested that research participants expect control over data sharing, and participants indicated that having control over with whom data is shared was more important than deciding what data types are shared. Page et al. (2016) also assessed the opinions of the patients regarding the research use of their health information and found that a substantial proportion of people indicated they did not need to know the exact research purpose or had no opinion on it.

Given that this project is a long-term study, establishing continuous relationships with participants is important in terms of maintaining the right direction of this project as a public research platform for the public good. Therefore, how to embed public opinion into the governance of the NPBBD is a concern that should be addressed. For example, although most respondents wished to receive information on incidental findings, there should be further discussion with stakeholders on the potential alternatives and the consequences of each option. In particular, limitations and challenges of lay understanding of genetic risk information should be considered (Wöhlke and Perry, 2021; Wöhlke et al., 2020; Ballard et al., 2020; Lewis et al., 2020; Hylind et al., 2018; Parry and Middleton, 2017; D’Abramo et al., 2015; Takashima et al., 2018). Furthermore, the perceived risks and benefits of data sharing should be discussed in the public forums to maintain a balance between making the best use of this platform and respecting the privacy preferences of the participants.

## 5 Limitations

This survey had two major limitations that should be addressed in future research. First, this exploratory online survey may not reflect the attitudes of the general population of South Korea. Some respondents may not have had enough time to contemplate the benefits and risks of participating in the project. This limitation could be overcome by qualitative studies, such as interviews, to investigate the understanding and concerns of the respondents in detail. Second, due to the nature of the survey, the respondents were more familiar with online settings than the average population. To a certain extent, this limitation was inevitable as the survey was conducted online. Nevertheless, as the survey was conducted on a large scale, the analyzed results could provide approximate perceptions, despite the limitations regarding the selection of respondents.

## 6 Conclusion

The results of this survey will guide the government in formulating integrated bio-big data projects and introducing related policies. First, we confirmed that there was a general consensus on the necessity and importance of this project. Second, expectations were mostly concerned with learning more about health conditions and improving health services. Meanwhile, no significant changes were observed in the major concerns regarding this project, such as the risk of data leakage and discrimination. Therefore, appropriate vigilance is an essential element to maintain trust among all stakeholders, and the security level of the data platform should be maintained with utmost care. Finally, more comprehensive consideration should be given to designing the return of the results scheme and embedding a public engagement model in this project. To this end, the ELSI researchers should strive to facilitate continuous communication between project operators, authorities, participants, researchers, and the public.

## Data Availability

The raw data supporting the conclusion of this article will be made available by the authors, without undue reservation.
