# Supplementary material for: Public perceptions and attitudes of the national project of bio-big data: A nationwide survey in the Republic of Korea
Source: Front Genet. 2023 Feb 23;14:1081812. doi: 10.3389/fgene.2023.1081812 (PMC9995590; doi:10.3389/fgene.2023.1081812)
Supplement: Supplementary file 1 [file Table1.docx]

Supplementary Material

**Supplementary Table S1** Multiple response cross-tabulation results of the positive factors affecting the decision to participate in the NPBBD. Participants were asked to indicate the three most positive variables on participation. Percentage and numbers are based on the total number of respondents. ^1^ 0.4% missing values. ^a^ Heard of it and know it well; ^b^ Heard of it but don't know it well; ^c^ Have not heard of it.

| Variable | Category | Contributing to the promotion of personalized medical services | Contributing to the competitiveness of the Korean bioindustry | Contributing to identifying the causes of cancer and rare diseases | Receiving healthcare information | Recommendations from medical personnel, such as attending physician | Recommendations from family members | Interest in government-driven projects | Other^1^ | Total |
| --- | --- | --- | --- | --- | --- | --- | --- | --- | --- | --- |
|  |  | N (%) | N (%) | N (%) | N (%) | N (%) | N (%) | N (%) | N (%) | N |
| All |  | 645(64.5) | 367(36.7) | 657(65.7) | 752(75.2) | 259(25.9) | 143(14.3) | 173(17.3) | 4(0.4) | 3000 |
| NPBBD Knowledge | Well^a^ | 65(58.0) | 44(39.3) | 65(58.0) | 76(67.9) | 31(27.7) | 25(22.3) | 30(26.8) | 0(0.0) | 336 |
|  | Moderate^b^ | 281(62.2) | 186(41.2) | 291(64.4) | 330(73.0) | 118(26.1) | 58(12.8) | 91(20.1) | 1(0.2) | 1356 |
|  | Not at all^c^ | 299(68.6) | 137(31.4) | 301(69.0) | 346(79.4) | 110(25.2) | 60(13.8) | 52(11.9) | 3(0.7) | 1308 |
| NPBBD participation | Willing | 379(65.5) | 233(40.2) | 394(68.0) | 431(74.4) | 131(22.6) | 66(11.4) | 102(17.6) | 1(0.2) | 1737 |
|  | Unsure | 235(64.4) | 115(31.5) | 225(61.6) | 279(76.4) | 113(31.0) | 66(18.1) | 61(16.7) | 1(0.3) | 1095 |
|  | Unwilling | 31(55.4) | 19(33.9) | 38(67.9) | 42(75.0) | 15(26.8) | 11(19.6) | 10(17.9) | 2(3.6) | 168 |
| Gender | Male | 324(63.0) | 204(39.7) | 339(66.0) | 363(70.6) | 137(26.7) | 74(14.4) | 97(18.9) | 4(0.8) | 1542 |
|  | Female | 321(66.0) | 163(33.5) | 318(65.4) | 389(80.0) | 122(25.1) | 69(14.2) | 76(15.6) | 0(0.0) | 1458 |
| Age | 20–29 | 133(59.4) | 99(44.2) | 137(61.2) | 153(68.3) | 62(27.7) | 31(13.8) | 56(25.0) | 1(0.4) | 672 |
|  | 30–39 | 144(64.6) | 79(35.4) | 123(55.2) | 168(75.3) | 72(32.3) | 47(21.1) | 36(16.1) | 0(0.0) | 669 |
|  | 40–49 | 176(64.7) | 90(33.1) | 187(68.8) | 216(79.4) | 68(25.0) | 30(11.0) | 47(17.3) | 2(0.7) | 816 |
|  | 50–59 | 192(68.3) | 99(35.2) | 210(74.7) | 215(76.5) | 57(20.3) | 35(12.5) | 34(12.1) | 1(0.4) | 843 |
| Education | high school | 94(69.6) | 45(33.3) | 92(68.1) | 106(78.5) | 28(20.7) | 24(17.8) | 15(11.1) | 1(0.7) | 405 |
|  | College | 478(63.2) | 281(37.2) | 495(65.5) | 563(74.5) | 207(27.4) | 105(13.9) | 136(18.0) | 3(0.4) | 2268 |
|  | Graduate school and beyond | 73(67.0) | 41(37.6) | 70(64.2) | 83(76.1) | 24(22.0) | 14(12.8) | 22(20.2) | 0(0.0) | 327 |

**Supplementary Table S2** Multiple response cross-tabulation results of the negative factors affecting the decision to participate in the NPBBD. Participants were asked to indicate the three most concerning factors. Percentage and numbers are based on the total number of respondents. ^1^0.5% missing values. ^a^ Heard of it and know it well; ^b^ Heard of it but don't know it well; ^c^ Have not heard of it.

| Variable | Category | Lack of time | Lack of information or consultation on the project | Possibility of knowing the risk of being diagnosed with an incurable disease | Data leakage | Discrimination | The possibility of using data in the industry | Opposition from family members | Other^1^ | Total |
| --- | --- | --- | --- | --- | --- | --- | --- | --- | --- | --- |
|  |  | N (%) | N (%) | N (%) | N (%) | N (%) | N (%) | N (%) | N (%) | N |
| All |  | 339 (33.9) | 406 (40.6) | 375 (37.5) | 685 (68.5) | 632 (63.2) | 339 (33.9) | 219 (21.9) | 5 (0.5) | 3000 |
| NPBBD Knowledge | Well^a^ | 43 (38.4) | 43 (38.4) | 46 (41.1) | 69 (61.6) | 63 (56.3) | 49 (43.8) | 22 (19.6) | 1 (0.9) | 336 |
|  | Moderate^b^ | 152 (33.6) | 185 (40.9) | 179 (39.6) | 302 (66.8) | 282 (62.4) | 150 (33.2) | 105 (23.2) | 1 (0.2) | 1356 |
|  | Not at all^c^ | 144 (33.0) | 178 (40.8) | 150 (34.4) | 314 (72.0) | 287 (65.8) | 140 (32.1) | 92 (21.1) | 3 (0.7) | 1308 |
| NPBBD participation | Willing | 204 (35.2) | 239 (41.3) | 216 (37.3) | 400 (69.1) | 365 (63.0) | 188 (32.5) | 122 (21.1) | 3 (0.5) | 1737 |
|  | Unsure | 116 (31.8) | 146 (40.0) | 144 (39.5) | 248 (67.9) | 232 (63.6) | 124 (34.0) | 84 (23.0) | 1 (0.3) | 1095 |
|  | Unwilling | 19 (33.9) | 21 (37.5) | 15 (26.8) | 37 (66.1) | 35 (62.5) | 27 (48.2) | 13 (23.2) | 1 (1.8) | 168 |
| Gender | Male | 189 (36.8) | 210 (40.9) | 189 (36.8) | 339 (66.0) | 314 (61.1) | 182 (35.4) | 116 (22.6) | 3 (0.6) | 1542 |
|  | Female | 150 (30.9) | 196 (40.3) | 186 (38.3) | 346 (71.2) | 318 (65.4) | 157 (32.3) | 103 (21.2) | 2 (0.4) | 1458 |
| Age | 20–29 | 85 (37.9) | 92 (41.1) | 84 (37.5) | 152 (67.9) | 136 (60.7) | 80 (35.7) | 43 (19.2) | 0 (0.0) | 672 |
|  | 30–39 | 83 (37.2) | 103 (46.2) | 80 (35.9) | 141 (63.2) | 139 (62.3) | 76 (34.1) | 47 (21.1) | 0 (0.0) | 669 |
|  | 40–49 | 91 (33.5) | 100 (36.8) | 101 (37.1) | 183 (67.3) | 171 (62.9) | 91 (33.5) | 76 (27.9) | 3 (1.1) | 816 |
|  | 50–59 | 80 (28.5) | 111 (39.5) | 110 (39.1) | 209 (74.4) | 186 (66.2) | 92 (32.7) | 53 (18.9) | 2 (0.7) | 843 |
| Education | High school | 37 (27.4) | 66 (48.9) | 59 (43.7) | 90 (66.7) | 83 (61.5) | 35 (25.9) | 35 (25.9) | 0 (0.0) | 405 |
|  | College | 263 (34.8) | 298 (39.4) | 281 (37.2) | 520 (68.8) | 482 (63.8) | 259 (34.3) | 162 (21.4) | 3 (0.4) | 2268 |
|  | Graduate school and beyond | 39 (35.8) | 42 (38.5) | 35 (32.1) | 75 (68.8) | 67 (61.5) | 45 (41.3) | 22 (20.2) | 2 (1.8) | 327 |
