# Supplementary material for: Public perceptions and attitudes of the national project of bio-big data: A nationwide survey in the Republic of Korea
Source: Front Genet. 2023 Feb 23;14:1081812. doi: 10.3389/fgene.2023.1081812 (PMC9995590; doi:10.3389/fgene.2023.1081812)
Supplement: Supplementary file 2 [file DataSheet1.pdf]

frontiers

# *Supplementary Material*

# Questionnaire

**Title: A Survey on Public Perception toward the Korean National Bio-Big Data Project**

## 1 Eligibility of participants

**SQ1. What is your gender?**

1. Male
2. Female

**SQ2. How old are you?**

1. < 19 years
2. 20–39 years
3. 40−49 years
4. 50−59 years
5. > 60 years

**SQ3. Where do you live?**

1. Seoul
2. Incheon
3. Busan
4. Daejeon
5. Daegu
6. Kwangju
7. Ulsan
8. Gyeonggi
9. Gangwon
10. Chungcheong
11. Gyeongsang
12. Jeolla
13. Jeju
14. Sejong

## 2 Part A. Awareness and expectations toward the project

**A1. Have you heard of 'bio-data' or 'big data in healthcare?**

1. I have heard of it and I know it well
2. I have heard of it but I don't know it well
3. I have not heard of it

**A2. Have you heard of a 'biobank"?**

1. I have heard of it and I know it well
2. I have heard of it but I don't know it well
3. I have not heard of it

**A3. Have you heard of the National project of Bio-Big Data?**

The National Project of Bio-Big Data(NPBBD) is designed to build a research resource platform that will become the basis for the development of preventive and personalized medicine. It aims to collect specific data such as clinical information, health insurance information, genomic data, and lifelogs (heart rate, footsteps, etc.) for a total of 1 million participants, including 400,000 patients with conditions such as rare diseases, cancer, severe incurable disease, and 600,000 healthy individuals. The collected data will be provided to researchers in a secure environment. The United States of America, the United Kingdom, and Finland are also involved in similar projects that utilize bio-big data resources to improve diagnosis and treatment and develop new innovative drugs and medical devices.

1. I have heard of it and I know it well (☞Go to A3-1)
2. I have heard of it but I don't know it well (☞Go to A3-1)
3. I have not heard of it (☞Go to A4)

**A3-1. How did you hear about the National project of Bio-Big Data? Please select all that apply.**

1. TV
2. Radio
3. Internet (social network services, blogs, etc.)
4. Newspapers/Magazines
5. Friends/family members
6. Billboards
7. Transit advertising
8. Guidance from the medical center (pamphlets or in-person introduction)
9. Education/Exhibition booth
10. Other

**A4. The following are the expected outcomes from implementing the NBBD. How important do you think this project is in obtaining each expected outcome?**

| Expected outcomes | Not important at all | Of little importance | Of average importance | Important | Very important |
|---|---|---|---|---|---|
| 1. Identification of the causes of cancer or rare diseases | 1 | 2 | 3 | 4 | 5 |
| 2. Promotion of personalized medicine based on the genetic data of Koreans | 1 | 2 | 3 | 4 | 5 |
| 3. Preventive medical services | 1 | 2 | 3 | 4 | 5 |
| 4. Extension of healthy quality of life years | 1 | 2 | 3 | 4 | 5 |
| 5. New drug development research innovations such as gene therapy | 1 | 2 | 3 | 4 | 5 |
| 6. Advancement of digital health devices | 1 | 2 | 3 | 4 | 5 |
| 7. Strengthening global competitiveness of the health industry | 1 | 2 | 3 | 4 | 5 |

**A5. If bio-big data is actively deployed in research through this project, how do you think the quality level of diagnosis and treatment will change compared to the present?**

1. It will be much worse
2. It will be somewhat worse
3. It will stay the same
4. It will be somewhat better
5. It will be much better

**Part B. Project participation decision-making factors**

**B1. If the NPBBD is implemented, would you be willing to participate?**

1. Definitely not
2. Probably not
3. Unsure
4. Probably yes
5. Definitely yes

**B2. With whom do you want to consult regarding participation in the NPBBD?**

1. None (I will decide on my own)
2. Medical personnel including my attending physician
3. NPBBD project staff
4. Family members
5. Friends
6. Other

**B3. From whom do you want to receive information regarding the NPBBD when deciding to participate in it?**

1. Medical personnel including my attending physician
2. NPBBD project staff
3. Family members
4. Friends
5. Other

**B4. What are the positive factors that would influence your potential decision to participate in the NPBBD? Please choose up to three of the following in the order which motivated you most.**

1. Contributing to the promotion of personalized medical services
2. Contributing to the Korean bioindustry's competitiveness
3. Contributing to identifying the causes of cancer and rare diseases
4. Receiving healthcare information
5. Recommendations from medical personnel, such as attending physician
6. Recommendations from family members
7. Interest in government-driven projects
8. Other

**B5. What are the negative factors that would influence your decision to participate in the NPBBD? Please choose up to three of the following in the order of your most concern.**

1. Lack of time
2. Lack of information or consultation on the NBBD
3. Possibility of knowing the risk of being diagnosed with an incurable diseases
4. Risk of data leakage
5. Discrimination concerns

6. Possibility of using data in the industry
7. Opposition from family members
8. Other

**Part C. Communication with participants and public engagement**

| |
|---|
| The National Project of Bio-Big Data stores and processes personal information permanently or semi-permanently (30 years) with the consent of participants. |

**C1. How would you like to receive information about the NPBBD? (e.g., information on the enrollment process, numbers of enrolled participants, data access criteria for researchers, research lists, the operation of the ethics committee, etc.)? Please select up to 2 methods in order of your preference.**

1. NPBBD website
2. Social media
3. Postal mail
4. Text messages or email
5. Phone calls

**C2. Would you be willing to use a website where you can check information about the NPBBD?**

1. Yes
2. No

**C3. Would you be interested in participating in the NPBBD committee made up of patients, their families, and the general public?**

1. Yes
2. No

**C4. What type of information would you like to receive regarding your personal test results from the NPBBD? Please select everything you want to know.**

1. Information related to pre-existing conditions/diseases
2. Information related to family history of diseases
3. Information on predicting disease risks based on genetic testing results
4. Incidental findings related to diseases that currently have few treatments available
5. Incidental findings related to diseases for which treatment is currently available
6. Healthcare information based on lifelogs (heart rate, footsteps)

**Part D. Scope of data sharing and utilization**

※ Please read the sentences in the box carefully and answer the following questions.

- Medical information refers to information related to the diagnosis, examination, and treatment of an individual's physical condition and disease.
- Whole genome sequencing (WGS) is a comprehensive method for analyzing 3 billion bases of the genome, revealing individual genetic differences, and providing information for diagnosis and customized treatment.
- To study the relationship between lifestyle and disease propensity and prognosis, biometric information or behavioral information such as heart rate changes, and the number of footsteps walked at a specific time can be used. These measures form the so-called lifelog.
- Wearable devices refer to electronic devices that are wearable on the body so that users can freely activate them while on the move or doing activities.
- All data collected are stored and processed with minimal individual identification by pseudonymizing information that can identify you.
- When providing data to researchers, we minimize the possibility of individual identification by pseudonymizing information that can identify you.

**D1. Would you like to share your medical information, including WGS, for each field of the study listed below?**

| Study fields | Yes | No | Not sure |
|---|---|---|---|
| 1. Cancer | 1 | 2 | 3 |
| 2. Rare disease | 1 | 2 | 3 |
| 3. Chronic diseases (e.g., diabetes, high blood pressure, etc.) | 1 | 2 | 3 |
| 4. Geriatric diseases (e.g., dementia, Parkinson's, stroke, etc.) | 1 | 2 | 3 |

**D2. Would you like to share your biometrics or behavioral information collected by smartphones or wearable devices, for each field of study listed below?**

| Study fields | Yes | No | Not sure |
|---|---|---|---|
| 1. Cancer | 1 | 2 | 3 |
| 2. Rare diseases | 1 | 2 | 3 |

| | | | |
|---|---|---|---|
| 3. Chronic diseases (e.g., diabetes, high blood pressure, etc.) | 1 | 2 | 3 |
| 4. Geriatric diseases (e.g., dementia, Parkinson's, stroke, etc.) | 1 | 2 | 3 |

**D3. Would you agree to share your medical information, including WGS, with each of the following entities/organizations for research? Please select all the items you would agree with.**

1. Government or public agencies
2. Government-funded research institutes
3. Private research institutes such as university hospitals
4. Non-profit organizations (e.g., patient advocacy groups)
5. Private pharmaceutical companies or medical device manufacturers
6. Individual researchers
7. Other
8. I have no intention of agreeing to share my medical information

**D4. Would you agree to share your biometrics or behavioral information collected from smartphones or wearable devices for research purposes with each of the following entities/organizations? Please select all the items you agree with.**

1. Government or public agencies
2. Government-funded research institutes
3. Private research institutes such as university hospitals
4. Non-profit organizations (e.g., patient advocacy groups)
5. Private pharmaceutical companies or medical device manufacturers
6. Individual researchers
7. Other
8. I have no intention of agreeing to share my medical information

**D5. Would you agree to share your medical information, including WGS, with each of the following overseas/international entities/organizations?**

1. International consortiums (e.g., ICGC, IRDiRC)
2. Foreign private research institutes such as university hospitals
3. Foreign private pharmaceutical companies or medical device manufacturers
4. Other
5. I have no intention of agreeing to share my medical information

**Part E. Healthcare-related services usage**

**E1. How often have you visited medical centers to check/treat your health? (Based on the last year)**

**E2. Do you have any private health insurance (excluding national health insurance)?**

1. Yes
2. No

**E3. Have you ever used a wearable device (e.g., Fitbit, Apple Watch, Galaxy Watch)?**

1. Yes
2. No

**E4. Have you ever used a healthcare app (e.g., Apple Health, Samsung Health, etc.)?**

1. Yes
2. No

**E5. Have you ever participated in a study that collects medical or genetic information?**

1. Yes
2. No

**DQ. Demographics**

**DQ1. What is your academic background?**

1. High school
2. College
3. Graduate school and more

**DQ2. What is your average monthly household income? Please respond based on the income of the entire family you live with (including investment income, bonuses, real estate income, etc.)**

1. < 2,000,000
2. 2,000,000 ~ 2,990,000
3. 3,000,000 ~ 3,990,000
4. 4,000,000 ~ 4,990,000
5. 5,000,000 ~ 5,990,000
6. 6,000,000 >
