# Supplementary material for: Public perceptions and attitudes of the national project of bio-big data: A nationwide survey in the Republic of Korea
Source: Front Genet. 2023 Feb 23;14:1081812. doi: 10.3389/fgene.2023.1081812 (PMC9995590; doi:10.3389/fgene.2023.1081812)
Supplement: Supplementary file 3 [file Image1.pdf]

Supplementary Material

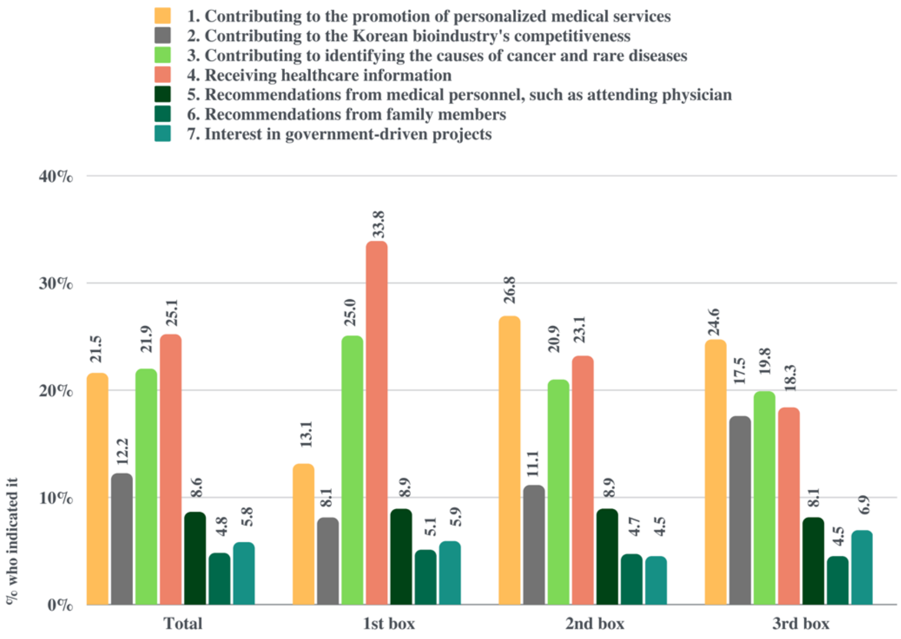

Supplementary Figure S1. Positive factors that would influence the decision to participate in the NPBBD. Participants were asked to indicate the three most positive factors in order. The percentage in the 'Total' category corresponds to the relative (n=100%) ratio of answered respponses across all three boxes.

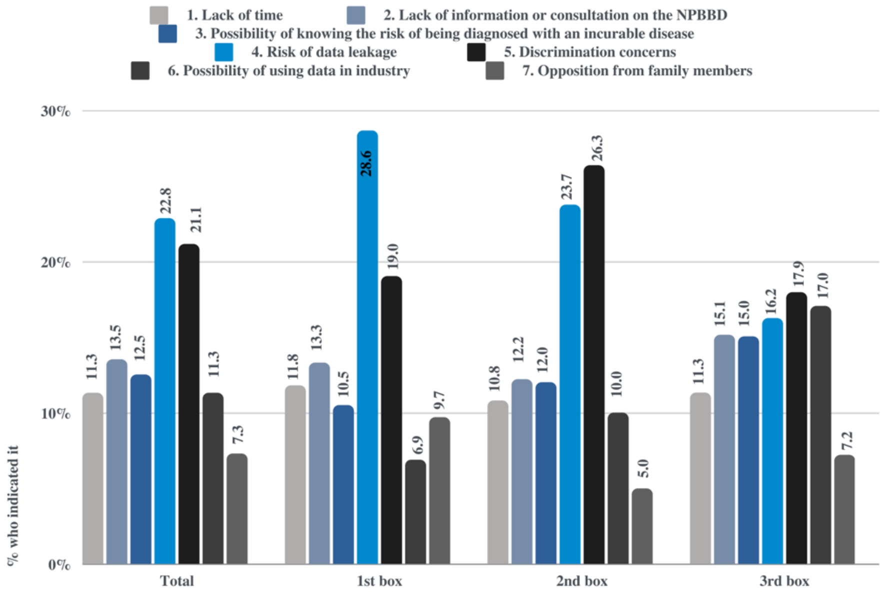

Supplementary Figure S2. Concerns that would affect the potential willingness to participate in the NPBBD. Participants were asked to indicate the three most negative factors in order. The percentage for the 'Total' category corresponds to the relative (n=100%) ratio of answered responses across all three boxes.

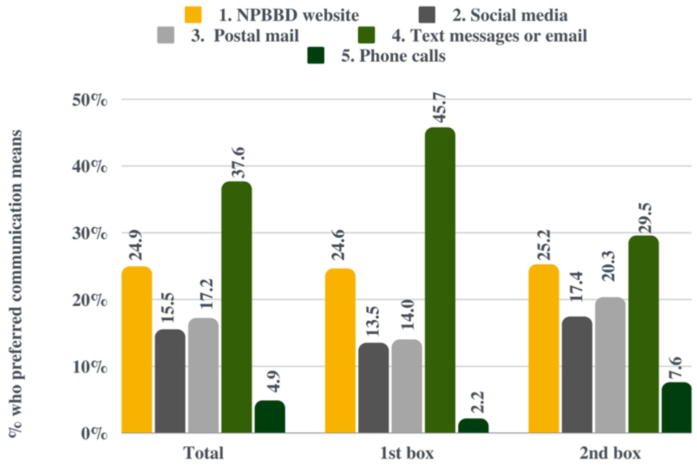

Supplementary Figure S3. Preferred ways to receive information about the NPBBD
